# Effects of statins and steroids on coronary artery disease and stroke in patients with interstitial lung disease and pulmonary fibrosis: A general population study

**DOI:** 10.1371/journal.pone.0259153

**Published:** 2021-10-27

**Authors:** Jun-Jun Yeh, Cheng-Li Lin, Nai-Hua Hsu, Chia-Hung Kao

**Affiliations:** 1 Ditmanson Medical Foundation Chia-Yi Christian Hospital, Chiayi, Taiwan; 2 China medical university, Taichung, Taiwan; 3 Management Office for Health Data, China Medical University Hospital, Taichung, Taiwan; 4 College of Medicine, China Medical University, Taichung, Taiwan; 5 Graduate Institute of Biomedical Sciences, College of Medicine, China Medical University, Taichung, Taiwan; 6 Department of Nuclear Medicine and PET Center, China Medical University Hospital, Taichung, Taiwan; 7 Department of Bioinformatics and Medical Engineering, Asia University, Taichung, Taiwan; 8 Center of Augmented Intelligence in Healthcare, China Medical University Hospital, Taichung, Taiwan; Universitaria di Bologna, ITALY

## Abstract

**Purpose:**

To determine the effects of statins and steroids on the risk of coronary artery disease (CAD) and stroke in patients with interstitial lung disease and pulmonary fibrosis (ILD-PF).

**Methods:**

We retrospectively enrolled patients with ILD-PF who were using statins (statin cohort, N = 11,567) and not using statins (nonstatin cohort, N = 26,159). Cox proportional regression was performed to analyze the cumulative incidence of CAD and stroke. Adjusted hazard ratios (aHRs) and 95% confidence intervals (CIs) of CAD and stroke were determined after sex, age, and comorbidities, as well as the use of inhaler corticosteroids (ICSs), oral steroids (OSs), and statins, were controlled for.

**Results:**

Compared with those of patients without statin use, the aHRs (95% CIs) of patients with statin use for CAD and ischemic stroke were 0.72 (0.65–0.79) and 0.52 (0.38–0.72), respectively. For patients taking single-use statins but not ICSs/OSs, the aHRs (95% CIs) for CAD and ischemic stroke were 0.72 (0.65–0.79)/0.69 (0.61–0.79) and 0.54 (0.39–0.74)/0.50 (0.32–0.79), respectively. For patients using ICSs/OSs, the aHRs (95% CIs) for CAD and ischemic stroke were 0.71 (0.42–1.18)/0.74 (0.64–0.85) and 0.23 (0.03–1.59)/0.54 (0.35–0.85), respectively.

**Conclusions:**

The findings demonstrate that statin use, either alone or in combination with OS use, plays an auxiliary role in the management of CAD and ischemic stroke in patients with ILD-PF.

## Introduction

Atherosclerosis is associated with system inflammation markers of interstitial lung disease (ILD) and pulmonary fibrosis (ILD-PF), including interleukin (IL)-6, IL-17, and the anti-inflammatory IL-10, as well as angiotensin-converting enzyme 2 (ACE2) [1–6]. Therefore, ILD-PF is considered an immunomodulatory and system inflammatory disease. Studies have reported that ILD-PF, particularly ILD-PF with acute exacerbation, increases the risks of coronary artery disease (CAD) and stroke. This is due to the natural course of ILD-PF, or concurrent infection [5,7,8]. Infections, including viral infections, may trigger cytokine storms; therefore, infections may be regarded as a contributor to hyperimmunity disorders. Moreover, they play a crucial role in CAD and stroke [9,10]. Taken together, the evidence indicates that ILD-PF with acute exacerbation increases the risks of these cardiovascular (CV) diseases in various scenarios.

Statins have an anti-inflammatory effect and have been found to prevent CAD and stroke [11,12]. Studies have observed that statins attenuate the inflammatory effect of IL-6 and IL-17 and modulate the anti-inflammatory effect of ACE2 in both ILD-PF and infection-induced pulmonary fibrosis [13,14], leading to reduced risks of CAD and stroke (S1 Appendix) [6,14–16]. These CV comorbidities have increased mortality among patients with ILD-PF during the ongoing virus pandemic. Thus, statin use in patients with ILD-PF merits further investigation. Herein, we determined whether statins attenuate the risk of CAD and stroke in patients with ILD-PF.

## Methods

### Data source

Taiwan’s single-payer National Health Insurance (NHI) program was established in 1995. This study used the Longitudinal Health Insurance Database, which contains the deidentified and encrypted medical claims data (for both inpatient and outpatient appointments) of 1 million beneficiaries randomly selected from the National Health Insurance Research Database (NHIRD). All diagnoses were made and recorded according to *International Classification of Diseases*, *9th Revision*, *Clinical Modification* (*ICD-9-CM*) codes.

#### Ethical approval and consent to participate

The NHIRD encrypts personal information to protect patients’ privacy. It provides researchers with anonymous identification numbers associated with relevant claims information, including sex, date of birth, medical services received, and prescriptions. Therefore, patient consent is not required to access the NHIRD. The study protocol was approved by the Institutional Review Board of China Medical University (CMUH104-REC2-115-AR4), which also specifically waived the informed consent requirement.

### Data and materials

The dataset used in this study is managed by Taiwan’s Ministry of Health and Welfare (MOHW). The MOHW approved our application to access these data. Any researcher interested in accessing this dataset must submit the relevant application to the MOHW. Please contact MOHW personnel (email: stcarolwu@mohw.gov.tw) for further assistance. MOHW address: No. 488, Sec. 6, Zhongxiao E. Rd., Nangang Dist., Taipei City 115, Taiwan (R.O.C.). Phone: +886-2-8590-6848. All relevant data are provided in this paper.

### Study population

The *ICD-9-CM* codes on the basis of which ILD-PF was diagnosed are provided in S2 Appendix. Patients having ILD-PF with infection, such as viral pneumonia (code 480), influenza (codes 487 and 488), and viral infection (079.0, 079.1, 079.2, 079.3, 079.4, 079.5, 079.6, and 079.8), or ILD-PF with respiratory failure (518.81, 518.82, 518.83, and 518.84) between 2000 and 2012 were enrolled [7,8,17–20]. The date of the first diagnosis of ILD-PF was set as the index date [18,20]. We excluded patients aged younger than 18 years and patients with a history of CAD or stroke before the index date, the *ICD-9-CM* codes of which are as follows: acute myocardial infarction (AMI), 410; other acute and subacute forms of ischemic heart disease, 411; old myocardial infarction, 412; angina pectoris, 413; other forms of chronic ischemic disease, 414; hemorrhagic stroke, 430–432; and ischemic stroke, 433–437. To assess the statin-associated risk of CAD and stroke, patients with ILD-PF were assigned to statin and nonstatin groups. The follow-up period ended on the date of a CAD or stroke event, date of death, date of withdrawal from the NHI, or December 31, 2013, whichever occurred first.

### Comorbidities and medications

Effects of comorbidities and medication were analyzed. Comorbidities correlated with the study endpoints comprised sleep disorders (codes 307.4 and 780.5), diabetes mellitus (code 250), hypertension (code 401–405), hyperlipidemia (code 272), alcohol-related illnesses (codes 291, 303, 305, 571.0, 571.1, 571.2, 571.3, and 790.3), chronic kidney disease (codes 585, 586, 588.8, and 588.9), gout (code 274), and cancer (codes 140–208). Furthermore, the effects of using inhaler corticosteroids (ICSs; e.g., budesonide/formoterol, fluticasone, and budesonide) were analyzed, as were those of using oral steroids (OSs; e.g., betamethasone, dexamethasone, methylprednisolone, triamcinolone, prednisone, prednisolone, hydrocortisone, and cortisone).

Atherosclerotic CV disease (ASCVD) included acute coronary syndromes, myocardial infarction, stable or unstable angina, arterial revascularization, stroke/transient ischemic stroke, and peripheral arterial diseases. However, a ≥20% 10-year ASCVD risk for a composite 3-point major atherosclerotic CV event of nonfatal myocardial infarction, nonfatal stroke, or CV death can serve as an arbitrary definition of patients with very high risk [21]. Thus, following the practice of the Taiwanese Secondary Prevention for Patients with Atherosclerotic Disease Registry [22,23], we replaced ASCVD with the following *ICD-9-CM* codes: AMI, 410; other acute and subacute forms of ischemic heart disease, 411; old myocardial infarction, 412; angina pectoris, 413; other forms of chronic ischemic disease, 414; hemorrhagic stroke, 430–432; ischemic stroke, 433–437; peripheral artery disease, 440.0, 440.2, 440.3, 440.8, 440.9, 443, 444.0, 444.22, 444.8, 447.8, and 447.9; arterial revascularization, 36.0–36.3; and operations on heart vessels, 36.9 [23,24]. The major atherosclerotic CV event was replaced with *ICD-9-CM* codes for all-cause mortality as follows: CV death, 390–459; nonfatal stroke, 430–438; nonfatal heart failure, 428; and AMI, 410. CV death was defined as a primary diagnosis of CV disease within 90 days of death.

### Statistical analysis

Descriptive statistical differences in demographic characteristics, comorbidities, and medication use between the statin and nonstatin groups were analyzed. The between-group differences in baseline distributions were examined through *t* tests for continuous variables and chi-square tests for categorical variables. The Kaplan–Meier method was employed to estimate the cumulative CAD-free and ischemic stroke–free survival rates, and the corresponding survival curves of both groups were plotted using a Cox model in which age, sex, comorbidities, and medication use were controlled for. Between-group differences were determined through a likelihood ratio test. Incidence rates of CAD and stroke (including ischemic and hemorrhagic stroke) were calculated for each group as the number of observed events divided by the total person-years of experience. Use of statins, namely atorvastatin, fluvastatin, lovastatin, pravastatin, rosuvastatin, and simvastatin, for a 6-month period or longer was quantified as a binary variable [25]. On account of the frequency of variations in statin use among patients with ILD during the study period, statins were considered time-dependent covariates in the Cox proportional hazards models. The Cox regression results were used to obtain the hazard ratios and 95% confidence intervals (CIs) of CAD and ischemic stroke in the statin and nonstatin groups. All adjusted hazard ratios (aHRs) and their 95% CIs were measured after the following were controlled for: age, sex, ICS use, OS use, and comorbidities of sleep disorders, diabetes, hypertension, hyperlipidemia, mental disorders, alcohol-related illnesses, chronic kidney disease, gout, and cancer. Analyses were performed using SAS software, Version 9.4 of the SAS System for Unix (SAS Institute Inc., Cary, NC, USA), and the figures were plotted in R software. Statistical significance was indicated if P < 0.05.

## Results

Among 37,726 patients with ILD-PF, 11,567 and 26,159 were in the statin and nonstatin groups, respectively (Table 1). The average ages of participants in the statin and nonstatin groups were 57.3 ± 12.5 and 47.6 ± 16.6 years, respectively. Statin nonusers were significantly younger than statin users (P < 0.001). Among the 37,726 patients, 56.8% were women. Statin users had significantly more greater comorbidities (P < 0.05) and significantly higher ICS and OS use (P < 0.05) compared with statin nonusers.

**Table 1:** Distributions of demographic and clinical comorbid status in study cohorts.

	Interstitial lung disease and pulmonary fibrosis						
	Statin						
	All(N = 37726)		No(N = 26159)		Yes(N = 11567)		
	n	%	n	%	n	%	p-value
**Age, years**							<0.001
<50	18364	48.7	15210	58.1	3154	27.3	
50–64	11887	31.5	6617	25.3	5270	45.6	
65+	7475	19.8	4332	16.6	3143	27.2	
Mean±SD ^a^	50.6	16.0	47.6	16.6	57.3	12.5	<0.001
**Gender**							<0.001
Women	21422	56.8	14989	57.3	6433	55.6	
Men	16304	43.2	11170	42.7	5134	44.4	
**Comorbidity**							
Sleep disorder	14700	39.0	9682	37.0	5018	49.4	<0.001
Diabetes	4805	12.7	1573	6.01	3232	27.9	<0.001
Hypertension	15564	41.3	7558	28.9	8006	69.2	<0.001
Hyperlipidemia	16612	44.0	6126	23.4	10486	90.7	<0.001
Mental disorders	17299	45.9	11170	42.7	6129	53.0	<0.001
Alcohol-related illness	3633	9.63	2391	9.14	1242	10.7	0.02
Chronic kidney disease	2008	5.32	797	3.05	1211	10.5	<0.001
Gout	6262	16.6	3026	11.6	3236	28.0	<0.001
Cancer	3965	10.5	2651	10.1	1314	11.4	<0.001
**Medication**							
Inhaled corticosteroids (ICSs)	1385	3.67	905	3.46	480	4.15	0.001
Oral steroids (OSs)	18500	49.0	12123	46.3	6377	55.1	<0.001

Chi-square test

^a^ t-test.

**Fig 1:**
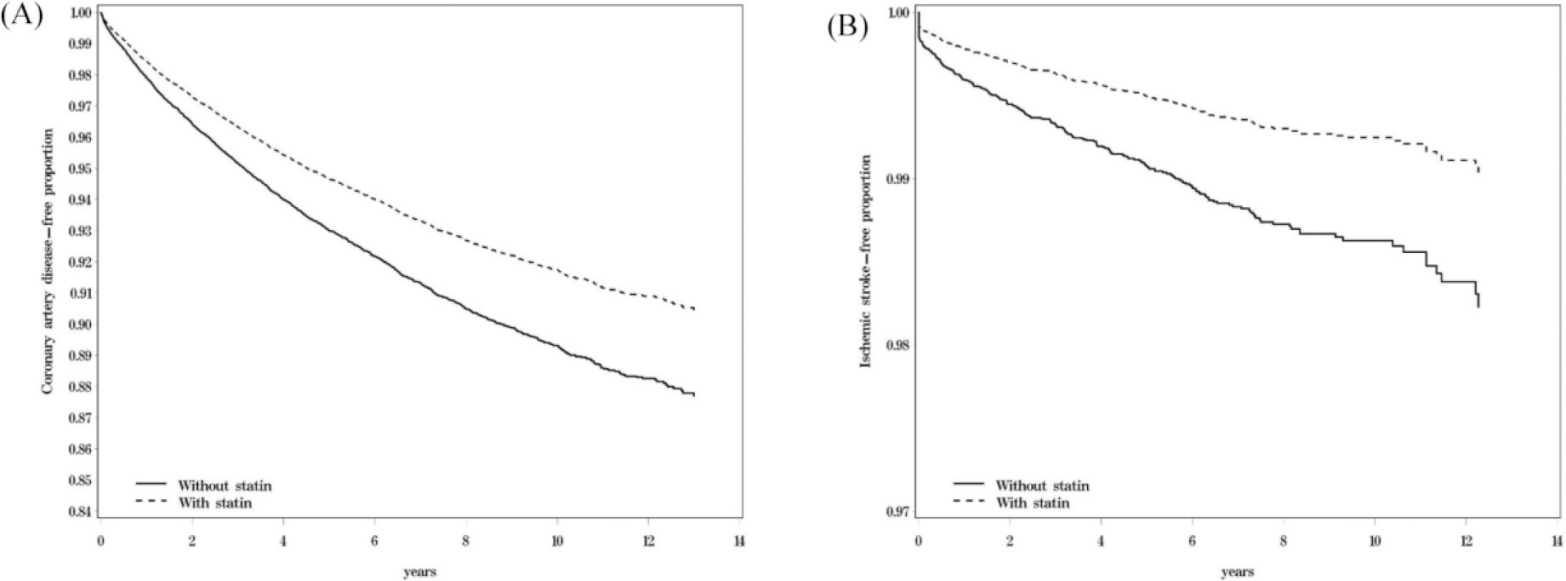
Coronary artery disease-free survival (A) and ischemic stroke-free survival (B) compared between patients with and without statin.

The statin group had lower cumulative CAD-free survival and ischemic stroke-free survival rates compared with the nonstatin group (Fig 1A and 1B). The incidence rates of CAD in the statin and nonstatin groups were 18.6 and 10.6 per 1000 person-years, respectively. Statin users had a lower risk of CAD (aHR = 0.72, 95% CI: 0.65–0.79) than did statin nonusers. The incidence rates of ischemic stroke in the statin and nonstatin groups were 1.32 and 1.47 per 1000 person-years, respectively. Statin users had a lower risk of ischemic stroke than did statin nonusers (aHR = 0.52, 95% CI: 0.38–0.72). Furthermore, the statin use cohort had a lower risk of ASCVD (aHR = 0.88, 95% CI: 0.82–0.95) than did the nonstatin cohort (Table 2).

**Table 2:** Overall Incidence of coronary artery disease and stroke (per 1,000 person-years) and estimated hazard ratios in interstitial lung disease and pulmonary fibrosis patients with statin compared to the interstitial lung disease and pulmonary fibrosis patients without statin by time dependent regression model.

	Statin		
Variables	No(N = 26159)	Yes(N = 11567)	p-value
**CAD**			
Person-years	129702	58716	
Follow-up time (y), Mean±SD	4.96±3.21	5.08±3.23	
Event, n	1372	1091	
Rate	10.6	18.6	
cHR (95% CI)	1(Reference)	1.76(1.63, 1.91)	<0.001
aHR (95% CI)^a^	1(Reference)	0.72(0.65, 0.79)	<0.001
**Stroke**			
Person-years	133747	60806	
Follow-up time (y), Mean±SD	5.11±3.27	5.26±3.30	
Event, n	859	786	
Rate	6.42	12.9	
cHR (95% CI)	1(Reference)	2.02(1.84, 2.23)	<0.001
aHR (95% CI)^a^	1(Reference)	1.00(0.88, 1.12)	0.83
**Ischemic stroke**			
Person-years	133747	60806	
Event, n	197	80	
Rate	1.47	1.32	
cHR (95% CI)	1(Reference)	0.90(0.70, 1.17)	0.98
aHR (95% CI)^a^	1(Reference)	0.52(0.38, 0.72)	0.001
**Hemorrhagic stroke**			
Person-years	133747	60806	
Event, n	662	706	
Rate	4.95	11.6	
cHR (95% CI)	1(Reference)	2.36(2.12, 2.62)	<0.001
aHR (95% CI)^a^	1(Reference)	1.12(0.98, 1.27)	0.10
**MACE**			
Person-years	125643	53351	
Event, n	553	618	
Rate	4.40	11.6	
cHR (95% CI)	1(Reference)	2.64(2.35, 2.96)	<0.001
aHR (95% CI)^a^	1(Reference)	1.14(0.99, 1.31)	0.08
**ASCVD**			
Person-years	116345	46867	
Event, n	2535	2157	
Rate	21.8	46.0	
cHR (95% CI)	1(Reference)	2.11(1.99, 2.23)	<0.001
aHR (95% CI)^a^	1(Reference)	0.88(0.82, 0.95)	<0.001

^a^Adjusting for age, gender, comorbidity of sleep disorder, diabetes, hypertension, hyperlipidemia, mental disorders, alcohol-related illness, chronic kidney disease, gout, and cancer, and medication of ICSs, and OSs.

Abbreviations: HD, cHR, crude hazard ratio; aHR, adjusted hazard ratio; ICSs, inhaled corticosteroids; OSs, oral steroids.

Stratified analyses were conducted on the basis of age (i.e., younger [<50 years] and older [≥50 years]) and sex. Statin users in all subgroups had a lower risk of CAD and ischemic stroke, excepting younger patients, who did not have a benefit of prevention of the ischemic stroke (Table 3). Regarding CAD risk, the aHRs (95% CIs) for men, women, younger patients, and older patients were 0.84 (0.74–0.96), 0.61 (0.53–0.70), 0.77 (0.63–0.94), and 0.68 (0.61–0.75), respectively. Regarding ischemic stroke risk, the aHRs (95% CIs) for men, women, younger patients, and older patients were 0.44 (0.29–0.69), 0.61 (0.38–0.98), 0.56 (0.26–1.18), and 0.51 (0.36–0.72), respectively. Furthermore, stratified analyses were conducted with the following subgroups: ICS nonusers, ICS users, OS nonusers, and OS users. Statin users in all subgroups, excepting ICS users, had a lower risk of CAD and ischemic stroke (Table 4) compared with statin nonusers. Regarding CAD risk, the aHRs (95% CIs) for ICS nonusers, OS users, and OS nonusers were 0.72 (0.65–0.79), 0.74 (0.64–0.85), and 0.69 (0.61–0.79), respectively. Regarding ischemic stroke risk, the aHRs (95% CIs) for ICS nonusers, OS users, and OS nonusers were 0.54 (0.39–0.74), 0.54 (0.35–0.85), and 0.50 (0.32–0.79), respectively.

**Table 3:** Overall Incidence (per 1,000 person-years) and hazard ratio for coronary artery disease and ischemic stroke in interstitial lung disease patients and pulmonary fibrosis with statin compared to the interstitial lung disease and pulmonary fibrosis patients without statin stratify by gender and age by time dependent regression model.

	**Statin**					
	**Men**			**Women**		
**Variable**	**No(N = 11170)**	**Yes(N = 5134)**	**p-value**	**No(N = 14989)**	**Yes(N = 6433)**	**p-value**
CAD						
No. of event	680	547		692	544	
Incidence rate	12.7	21.8		9.11	16.2	
cHR (95% CI)	1(Reference)	1.72(1.54, 1.93)	<0.001	1(Reference)	1.78(1.59, 2.00)	<0.001
aHR (95% CI)^a^	1(Reference)	0.84(0.74, 0.96)	0.009	1(Reference)	0.61(0.53, 0.70)	<0.001
Ischemic stroke						
No. of event	127	36		70	44	
Incidence rate	2.29	1.38		0.89	1.27	
cHR (95% CI)	1(Reference)	0.61(0.42, 0.88)	0.008	1(Reference)	1.43(0.98, 2.08)	0.06
aHR (95% CI)^a^	1(Reference)	0.44(0.29, 0.69)	<0.001	1(Reference)	0.61(0.38, 0.98)	0.04
	**Statin**					
	**Age<50**			**Age≧50**		
	**No(N = 15210)**	**Yes(N = 3154)**		**No(N = 10949)**	**Yes(N = 8413)**	
CAD						
No. of event	370	236		1002	855	
Incidence rate	4.50	12.7		21.1	21.3	
cHR (95% CI)	1(Reference)	2.83(2.40, 3.33)	<0.001	1(Reference)	1.02(0.93, 1.12)	0.71
aHR (95% CI)^a^	1(Reference)	0.77(0.63, 0.94)	0.009	1(Reference)	0.68(0.61, 0.75)	<0.001
Ischemic stroke						
No. of event	55	14		142	66	
Incidence rate	0.66	0.73		2.83	1.59	
cHR (95% CI)	1(Reference)	1.15(0.64, 2.08)	0.63	1(Reference)	0.57(0.43, 0.77)	<0.001
aHR (95% CI)^a^	1(Reference)	0.56(0.26, 1.18)	0.12	1(Reference)	0.51(0.36, 0.72)	<0.001

^a^Adjusting for comorbidity of sleep disorder, diabetes, hypertension, hyperlipidemia, mental disorders, alcohol-related illness, chronic kidney disease, gout, and cancer, and medication of ICSs, and OSs.

Abbreviations: HD, cHR, crude hazard ratio; aHR, adjusted hazard ratio; ICSs, inhaled corticosteroids; OSs, oral steroids.

**Table 4:** Overall Incidence (per 1,000 person-years) and hazard ratio for coronary artery disease and ischemic stroke in interstitial lung disease and pulmonary fibrosis patients with statin compared to the interstitial lung disease and pulmonary fibrosis patients without statin by ICSs status and OSs status by time dependent regression model.

	**Statin**					
	**With ICSs**			**Without ICSs**		
	**No(N = 905)**	**Yes(N = 480)**	**p-value**	**No(N = 25254)**	**Yes(N = 11087)**	**p-value**
CAD						
No. of event	52	40		1320	1051	
Incidence rate	11.3	14.4		10.6	18.8	
cHR (95% CI)	1(Reference)	1.28(0.85, 1.93)	0.24	1(Reference)	1.78(1.65, 1.94)	<0.001
aHR (95% CI)^a^	1(Reference)	0.71(0.42, 1.18)	0.19	1(Reference)	0.72(0.65, 0.79)	<0.001
Ischemic Stroke						
No. of event	7	2		190	78	
Incidence rate	1.49	0.70		1.47	1.35	
cHR (95% CI)	1(Reference)	0.49(0.10, 2.36)	0.37	1(Reference)	0.92(0.71, 1.20)	0.54
aHR (95% CI)^a^	1(Reference)	0.23(0.03, 1.59)	0.14	1(Reference)	0.54(0.39, 0.74)	<0.001
	**Statin**					
	**With OSs**			**Without OSs**		
	**No(N = 12123)**	**Yes(N = 6377)**		**No(N = 14036)**	**Yes(N = 5190)**	
CAD						
No. of event	629	535		743	556	
Incidence rate	10.2	15.9		10.9	22.2	
cHR (95% CI)	1(Reference)	1.55(1.39, 1.74)	<0.001	1(Reference)	2.04(1.83, 2.27)	<0.001
aHR (95% CI)^a^	1(Reference)	0.74(0.64, 0.85)	<0.001	1(Reference)	0.69(0.61, 0.79)	<0.001
Ischemic Stroke						
No. of event	89	43		108	37	
Incidence rate	1.41	1.24		1.53	1.42	
cHR (95% CI)	1(Reference)	0.89(0.62, 1.27)	0.51	1(Reference)	0.93(0.64, 1.35)	0.69
aHR (95% CI)^a^	1(Reference)	0.54(0.35, 0.85)	0.007	1(Reference)	0.50(0.32, 0.79)	0.003

^a^Adjusting for age, gender, comorbidity of sleep disorder, diabetes, hypertension, hyperlipidemia, mental disorders, alcohol-related illness, chronic kidney disease, gout, and cancer, and medication of ICSs, and OSs.

Abbreviations: HD, cHR, crude hazard ratio; aHR, adjusted hazard ratio; ICSs, inhaled corticosteroids; OSs, oral steroids.

### Validation of interstitial lung disease, pulmonary fibrosis, stroke, and CAD

Studies outside Taiwan using administrative and claims data have applied validated methods for identifying ILD-PF; one study, for example, determined that *ICD-9-CM* codes 515 and 516.3 indicated idiopathic pulmonary fibrosis–pulmonary fibrosis [18,26]. Regardless of the diagnostic codes used for ILD-PF identification, such algorithms can be improved using procedural codes, such as those for lung biopsies and relevant imaging techniques (e.g., high-resolution computed tomography) [19]. Studies in Taiwan have diagnosed ILD-PF on the basis of these criteria [20,26,27].

A study in Taiwan used the Patients with Catastrophic Illnesses or Rare Diseases database to enroll patients with autoimmune diseases, such as rheumatoid lung disease [28]; systemic lupus erythematosus; multiple sclerosis; Sjögren’s syndrome; cancer, occupational lung diseases; and postinflammatory pulmonary fibrosis, alveolar pneumonopathy, or idiopathic pulmonary fibrosis [8,18]. Data on viral pneumonia, influenza, and viral chlamydia infection combined with ILD-PF are included in this database, which is a subsection of the NHIRD [8,29–31]. All registrants with ILD-PF or respiratory failure are given catastrophic illness certificates on the basis of clinical and laboratory diagnoses by rheumatologists or pulmonologists [31]. The NHI program waives copayments for ILD-related treatments for these registrants. The records of all patients identified as having ILD-PF according to our classification criteria were reviewed by a pulmonologist and a rheumatologist [32]. In Taiwan, protocols concerning the diagnosis of CAD and stroke are well established. In this context, CAD encompasses angina, coronary intervention, coronary surgery, and death from myocardial infarction. CAD and stroke were diagnosed according to clinical data, imaging results (e.g., angiography), or surgical intervention [23,33]. These risks have been validated in previous studies [23,32]. The validation of CAD and stroke in the NHIRD revealed that their positive predictive values were high—up to 88% and 88.4% for CAD and ischemic stroke, respectively [23,33].

### Sensitivity analysis

We stratified the study cohort on the basis of sex, age (<50 and ≥50 years), statin (ICS/OS) use, and statin use without ICS or OS use. Statin users in all subgroups, except for the younger subgroup, had a lower risk of ischemic stroke than did statin nonusers. Moreover, statin users in all subgroups had a lower risk of CAD and ischemic stroke, except in those with combined statin and ICS use. These findings are consistent with the primary outcome. The two populations differed in size and baseline characteristics; thus, we conducted time-dependent and subgroup analyses [34]. For example, the older subgroup (statin use: statin nonuse, n = 8413:10,949) had lower risks of CAD and ischemic stroke. Moreover, after baseline cofounders were adjusted, independent risk factors between the two groups were identified, revealing that the statin cohort had a lower risk of ASCVD than the nonstatin cohort.

## Discussion

The most essential finding of this study is that statins lowered the risks of CAD and ischemic stroke among patients with ILD-PF, regardless of age, sex, and comorbidities. Furthermore, the combined use of statins and OS/ICS use lowered these risks. In line with our results, Vedel-Krogh et al. reported that statin use reduced mortality rates in patients with ILD-PF [35].

The most serious pulmonary complication from connective tissue disorders is the involvement of blood vessels in the lungs, which reduces oxygen uptake and causes pulmonary arterial hypertension (i.e., increased blood pressure in the pulmonary arteries). Hypoxemia is associated with atherosclerosis. Similar to idiopathic pulmonary fibrosis, collagen vascular diseases (e.g., lupus, Sjögren’s syndrome, and dermatomyositis) increase the risk of simultaneous hypoxemia and respiratory failure. Therefore, the presence of collagen vascular diseases and ILD-PF increases the risks of atherosclerotic diseases, such as CAD and ischemic stroke [25]. Statins attenuate IL-6 and enhance ACE2, thereby mitigating the risk of CAD and ischemic stroke. In accordance with our findings, in a prospective trial on cardiac protection, statins were found to reduce IL-6-induced C-reactive protein levels in the final year of follow-up [36].

Steroids may attenuate pulmonary fibrosis through the modulation of transforming growth factor beta-3 in patients with ILD-PF and infection [37], thereby reducing hypoxemia. Thus, steroid use can reduce hypoxic atherosclerosis (e.g., stroke), particularly in cases of viral infection with silent hypoxemia. [38] Herein, we observed that both OS use and OS–statin use reduced the risk of CAD and stroke. However, steroid and statin use in ILD-PF warrants further exploration in large-scale studies.

Our findings indicate that statins play an auxiliary role in the management of CAD and ischemic stroke among patients with ILD-PF (e.g., systemic sclerosis and sarcoidosis, *ICD-9-CM* codes 710.1 and 135, respectively) [39,40] with acute exacerbation. This is due to the natural course of ILD-PF, or concurrent infection.

### Strengths

The strength of this study is that our data source was a large population-based database under a universal health-care system. Furthermore, we employed validated algorithms for the determination of baseline comorbidities and confirmation of ILD-PF diagnoses.

Considering that adherence to statin treatment was suboptimal in practice, we performed a time-dependent analysis to prevent bias related to the confounding factors of nonadherence from affecting the results. We replaced hypertension, hyperlipidemia, and diabetes with obesity; alcohol-related disease with lifestyle; ICS and OS use with chronic obstructive pulmonary disease; and smoking, air pollution, and mental and sleep disorders with adherence. Furthermore, we stratified the patients by age and sex for sensitivity analysis, the results of which accord with our principal findings.

### Limitations

Our study has several limitations. First, as with all studies that use administrative data, unmeasured confounders may be present. These confounders include the use of over-the-counter medications (e.g., nonsteroidal anti-inflammatory drugs), intercurrent illness, and the underlying risk of CAD and stroke. Second, the NHIRD does not contain data on blood pressure, blood glucose, cholesterol, renal function, or liver function, which are useful for assessing CAD and stroke status. However, the inclusion of such data is unlikely to change our key findings, although it may reduce our findings’ statistical power due to effect modification by baseline CAD and stroke status. Notably, low estimated glomerular filtration rate does not greatly affect blood statin levels because these medications work through hepatic metabolism rather than renal excretion. Third, as is the case in most pharmacoepidemiological studies, we examined medication use according to data on the dispensation of medication from pharmacies. Some participants may have not taken the medications on the indicated dates, if at all. However, statins were considered time-dependent covariates in the present Cox proportional hazards models. Despite these limitations, the findings contribute to the management of ILD-PF for the prevention of CV diseases.

### Conclusion

Statin use, whether alone or in combination with OS use, plays an auxiliary role in the management of CAD and ischemic stroke in patients with ILD-PF. Large-scale follow-up studies on the use of steroids and statins in ILD-PF are required.

## Supporting information

S1 RECORD checklist The RECORD statement–checklist of items, extended from the STROBE statement, that should be reported in observational studies using routinely collected health data. (DOCX)

S1 Appendix Full name of ICD-9CM with interstitial lung disease and pulmonary fibrosis (ILD-PF). (TIF)

S2 Appendix Statins attenuate the inflammatory effect of IL-6 and IL-17 and modulate the anti-inflammatory effect of ACE2 in both ILD-PF and infection-induced pulmonary fibrosis, leading to reduced risks of CAD and stroke. (DOCX)

## Abbreviations

CAD: coronary artery disease
ILD: interstitial lung disease
aHR: adjusted hazard ratio
CI: confidence interval
ICS: inhaler corticosteroid
OS: oral steroids
IPF: idiopathic pulmonary fibrosis
ACE2: angiotension convergin enzyme 2
ICD-9-CM: International Classification of Diseases, Ninth revision, Clinical Modification
NHIRD: National Health Insurance Research Database
