# Supplementary figures and images for: Effects of statins and steroids on coronary artery disease and stroke in patients with interstitial lung disease and pulmonary fibrosis: A general population study
Source: PLoS One. 2021 Oct 27;16(10):e0259153. doi: 10.1371/journal.pone.0259153 (PMC8550436; doi:10.1371/journal.pone.0259153)

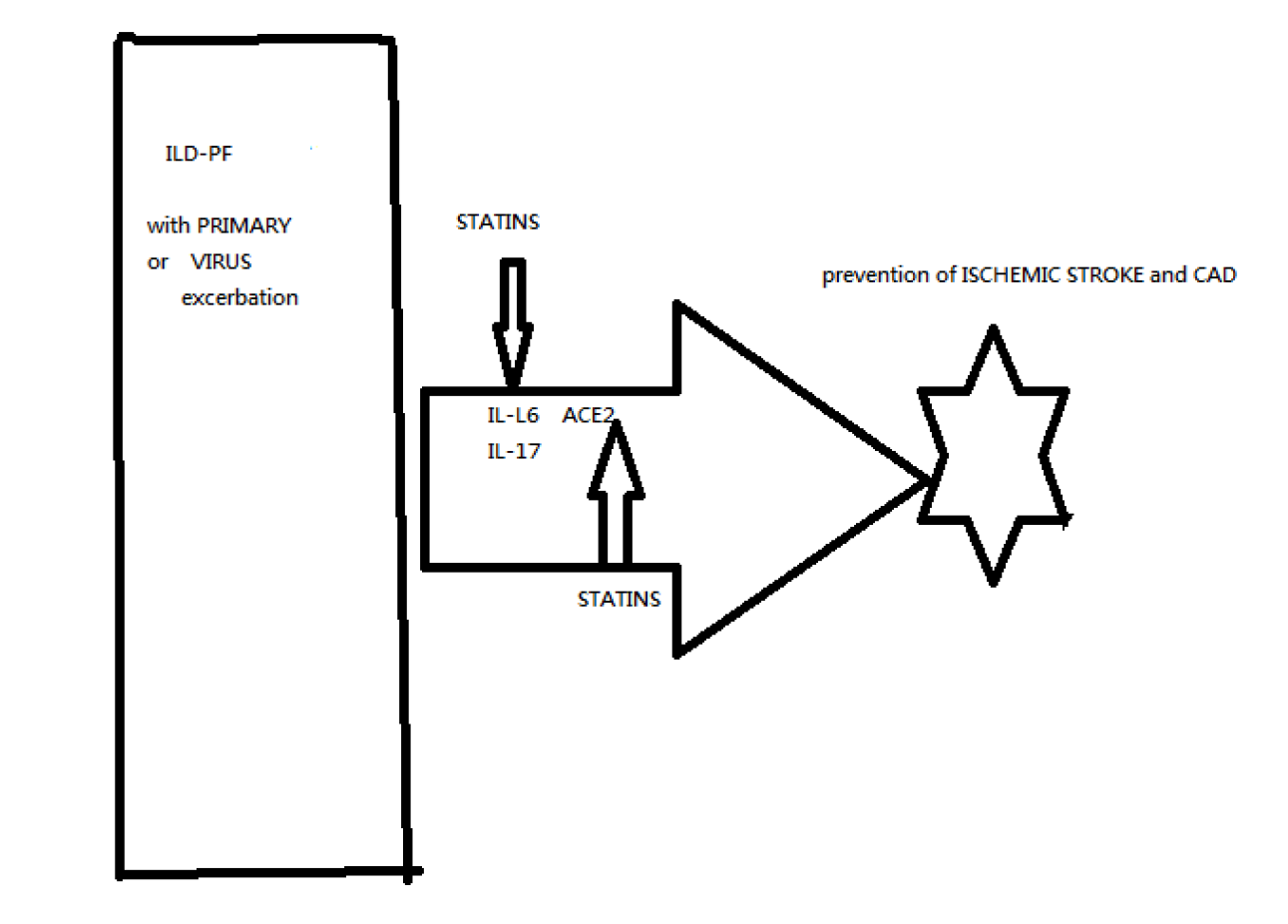

Supplement: S1 Appendix — (TIF) [file pone.0259153.s002.tif]
