# Supplementary material for: Effects of statins and steroids on coronary artery disease and stroke in patients with interstitial lung disease and pulmonary fibrosis: A general population study
Source: PLoS One. 2021 Oct 27;16(10):e0259153. doi: 10.1371/journal.pone.0259153 (PMC8550436; doi:10.1371/journal.pone.0259153)
Supplement: S2 Appendix — (DOCX) [file pone.0259153.s003.docx]

**Appendix Table. Full name of ICD-9CM with interstitial lung disease and pulmonary fibrosis (ILD-PF).**

| 135 | sarcoidosis |
| --- | --- |
| 237.7 | neurofibromatosis |
| 272.7 | lipidoses |
| 277.3 | amyloidosis |
| 277.8 | other specified disorders of metabolism (including eosinophilic granuloma) |
| 446.21 | Goodpasture's syndrome |
| 446.4 | Wegener's granulomatosis |
| 495 | extrinsic allergic alveolitis |
| 500 | coal workers’ pneumoconiosis |
| 501 | asbestosis |
| 502 | pneumoconiosis due to other silica or silicates |
| 503 | pneumoconiosis due to other inorganic dust |
| 504 | pneumonopathy due to inhalation of other dust |
| 505 | pneumoconiosis (unspecified) |
| 506.4 | chronic respiratory conditions due to chemicals, gases, fumes, and vapors |
| 508.1 | chronic and other pulmonary manifestations due to radiation |
| 508.8 | respiratory conditions due to other specified external agents |
| 515 | postinflammatory pulmonary fibrosis |
| 516 | other alveolar and parietoalveolar pneumonopathy, which includes |
| 516.30 | idiopathic interstitial pneumonia not otherwise specified |
| 516.31 | idiopathic pulmonary fibrosis |
| 516.32 | idiopathic nonspecific interstitial pneumonitis |
| 516.33 | acute interstitial pneumonitis |
| 516.34 | respiratory bronchiolitis interstitial lung disease |
| 516.35 | idiopathic lymphoid interstitial pneumonia |
| 516.36 | cryptogenic organizing pneumonia |
| 516.37 | desquamative interstitial pneumonia |
| 517.2 | lung involvement in systemic sclerosis |
| 517.8 | lung involvement in other diseases classified elsewhere; |
| 518.3 | pulmonary eosinophilia |
| 555 | Crohn's disease - |
| 710 | diffuse diseases of connective tissue |
| 710.1 | systemic sclerosis |
| 710.2 | Sjögren’s disease |
| 710.3 | dermatomyositis |

| 710.4 | polymyositis |
| --- | --- |
| 714.81 | rheumatoid lung |
| 720 | ankylosing spondylitis and other inflammatory spondylopathies |
| 759.5 | tuberous sclerosis |

- **The ILD-PF with virus pneumonia, Influenza, virus infection enrolled into the ILD-PF cohort.**
